# Supplementary material for: New Series of Zaxinone Mimics (MiZax) for Fundamental and Applied Research
Source: Biomolecules. 2023 Aug 1;13(8):1206. doi: 10.3390/biom13081206 (PMC10452442; doi:10.3390/biom13081206)
Supplement: Supplementary file 1 [file biomolecules-13-01206-s001.zip › Figure S1.pdf]

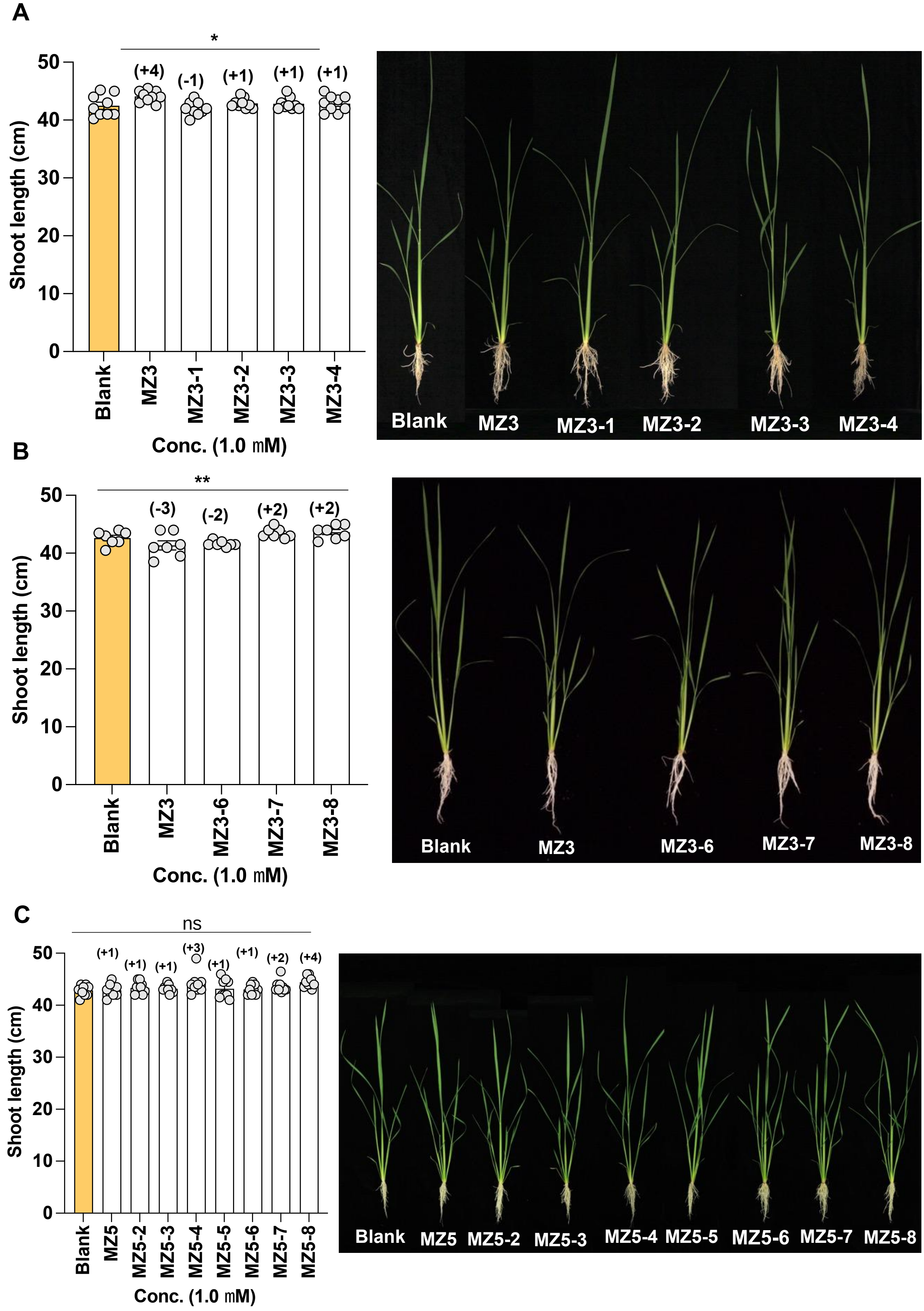

Figure S1. Effect of newly modified MZ3 and MZ5 derivatives on rice shoot growth. (A) The phenotype of shoot of rice seedlings in response to MZ3-1 to MZ3-4 application. (B) The phenotype of shoot of rice seedlings upon application of MZ3-6 to MZ3-8. (C) The phenotype of shoot of rice seedlings in response to MZ5-2 to MZ5-8. The newly modified compounds were applied at 1.0 µM to hydroponically grown rice seedlings for two weeks.
