# Supplementary material for: New Series of Zaxinone Mimics (MiZax) for Fundamental and Applied Research
Source: Biomolecules. 2023 Aug 1;13(8):1206. doi: 10.3390/biom13081206 (PMC10452442; doi:10.3390/biom13081206)
Supplement: Supplementary file 1 [file biomolecules-13-01206-s001.zip › Table S1.pdf]

Table S1: Physico-chemical properties of new MiZax3 and MiZax5 derivatives

MZ3-1	^{1}H-NMR (500 MHz, $CDCl_3$) δ: 7.22 (t, J = 7.5 Hz, 1H), 7.04 (d, J = 8.0 Hz, 1H), 6.93-7.00 (m, 3H), 6.87 (d, J = 9.0 Hz, 2H), 6.81 (d, J = 8.0 Hz, 1H) , 6.48 (d, J = 16.0 Hz, 1H) , 6.20 (dd, J = 16.0 Hz, 6.0 Hz, 1H) , 4.43 (m, 1H), 3.79 (s, 3H) , 1.33 (d, J = 6.5 Hz, 3H). ^{13}C-NMR (126 MHz, DMSO) δ: 158.87, 156.01, 150.13, 138.63, 134.39, 129.83, 128.88, 120.91 (2C), 120.89, 116.96, 115.49, 115.00 (2C), 68.79, 55.74, 23.48. HRMS (ESI) *m/z* $[M+H]^+$ calcd for $C_{17}H_{19}O_3$ 271.1329, found 271.1332. Yield 80**%**.
MZ3-2	^{1}H-NMR (500 MHz, $CDCl_3$) δ: 7.22 (t, J = 8.0 Hz, 1H), 7.07 (d, J = 7.5 Hz, 1H), 6.94-7.00 (m, 3H), 6.87 (d, J = 9.0 Hz, 2H), 6.82 (d, J = 8.0 Hz, 1H) , 6.46 (d, J = 16.0 Hz, 1H) , 6.04 (dd, J = 16.0 Hz, 7.0 Hz, 1H) , 3.85 (m, 1H), 3.78 (s, 3H), 3.29 (s, 3H), 1.30 (d, J = 6.5 Hz, 3H). ^{13}C-NMR (126 MHz, DMSO) δ: 158.72, 155.84, 149.87, 138.31, 132.04, 130.69, 129.62, 120.73, 120.63, 116.78, 115.27, 114.77, 77.85, 55.98, 55.48, 21.28. HRMS (ESI) *m/z* $[M+H]^+$ calcd for $C_{18}H_{21}O_3$ 285.1485, found 285.1483. Yield 80**%**.
MZ3-3	^{1}H-NMR (500 MHz, $CDCl_3$) δ: 7.25 (t, J = 8.0 Hz, 1H), 7.15 (d, J = 7.5 Hz, 1H), 7.00 (s, 1H), 6.98 (d, J = 9.0 Hz, 2H), 6.85-6.90 (m, 3H), 6.77 (d, J = 16.0 Hz, 1H), 6.73 (d, J = 16.0 Hz, 1H) , 3.93 (m, 3H), 3.79 (s, 3H) , 2.03 (s, 3H). ^{13}C-NMR (126 MHz, DMSO) δ: 158.95, 155.95, 155.43, 138.04, 132.36, 129.81, 126.37, 121.06, 120.88, 117.55, 115.07, 114.86, 61.79, 55.50, 10.03. E-configuration was confirmed by NOESY measurement. HRMS (ESI) *m/z* $[M+H]^+$ calcd for $C_{18}H_{20}NO_3$ 298.1438, found 298.1433. yield 66**%**
MZ3-4	^{1}H-NMR (500 MHz, CDCl3) δ: 7.42 (d, J = 16.5 Hz, 1H), 7.27 (t, J = 7.5 Hz, 1H), 7.20 (d, J = 7.5 Hz, 1H), 7.08 (s, 1H), 6.98 (d, J = 9.0 Hz, 2H), 6.86-6.91 (m, 3H), 6.82 (d, J = 16.5 Hz, 1H), 3.90 (s, 3H), 3.80 (s, 3H) , 2.08 (s, 3H). ^{13}C-NMR (126 MHz, DMSO) δ: 158.78, 155.94, 152.20, 149.88, 138.02, 135.59, 129.84, 121.49, 120.73, 188.14, 117.89, 116.49, 114.87, 61.55, 55.59, 16.84. Z-configuration was confirmed by NOESY measurement. HRMS (ESI) *m/z* $[M+H]^+$ calcd for $C_{18}H_{20}NO_3$ 298.1438, found 298.1435. yield 23**%**
MZ3-6	^{1}H-NMR (500 MHz, $CDCl_3$) δ: 7.48 (d, J = 16.5 Hz, 1H), 7.31 (t, J = 8.0 Hz, 1H), 7.22 (d, J = 8.0 Hz, 1H), 7.10 (s, 1H), 6.96-7.02 (m, 3H), 6.91 (d, J = 8.0 Hz, 2H), 6.66 (d, J = 16.5 Hz, 1H) , 3.82 (s, 3H) , 2.68 (q, J = 7.5 Hz, 2H), 1.16 (t, J = 7.5 Hz, 3H). ^{13}C-NMR (126 MHz, DMSO) δ: 200.80, 159.09, 156.18, 149.46, 141.61, 136.23, 130.07, 126.51, 122.41, 121.00 (2C), 119.38, 116.39, 114.97 (2C), 55.60, 33.96, 8.14. HRMS (ESI) *m/z* $[M+H]^+$ calcd for $C_{18}H_{19}O_3$ 283.1329, found 283.1324.
MZ3-7	^{1}H-NMR (500 MHz, CDCl3) δ:7.61 (d, J = 16.0 Hz, 1H), 7.30 (t, J = 8.0 Hz, 1H), 7.19 (d, J = 8.0 Hz, 1H), 7.06 (s, 1H), 6.65-7.01 (m, 3H), 6.90 (d, J = 9.0 Hz, 2H), 6.36 (d, J = 16.0 Hz, 1H), 3.82 (s, 3H), 3.79 (s, 3H).13C-NMR (126 MHz, DMSO) δ: 167.23, 159.10, 156.21, 149.44, 144.31, 135.98, 130.05, 122.26, 121.06 (2C), 119.30, 118.37, 116.25, 114.98 (2C), 55.63, 51.71 .HRMS (ESI) *m/z* $[M+H]^+$ calcd for $C_{17}H_{17}O_4$ 285.1121, found 285.1117.
MZ3-8	^{1}H-NMR (500 MHz, $CDCl_3$) δ:7.52 (d, J = 16.0 Hz, 1H), 7.31 (t, J = 7.5 Hz, 1H), 7.23 (d, J = 7.5 Hz, 1H), 7.12 (s, 1H), 6.94-7.00 (m, 3H), 6.90 (d, J = 9.0 Hz, 2H), 6.73 (d, J = 16.0 Hz, 1H), 3.82 (s, 3H), 2.92 (tt, J = 7.0 Hz, 1H), 1.16 (d, J = 7.0 Hz, 6H). ^{13}C-NMR (126 MHz, DMSO) δ: 203.69, 159.03, 156.16, 149.56, 141.80, 126.36, 130.04, 125.02, 122.46, 120.93 (2C), 119.35, 116.63, 114.97 (2C), 55.62, 39.15, 18.43 (2C) . HRMS (ESI) *m/z* $[M+H]^+$ calcd for $C_{19}H_{21}O_3$ 297.1485, found 297.1475.

MZ5-2	^{1}H-NMR (500 MHz, CDCl3) δ: 9.15 (d, J = 2.0 Hz, 1H), 9.07 (d, J = 2.0 Hz, 1H), 8.46 (d, J = 2.0 Hz, 1H), 7.77 (s, 1H), 7.65-7.60 (m, 1H), 7.58 (d, J = 9.0 Hz, 2H), 7.57-7.53 (m, 2H), 7.01 (d, J = 9.0 Hz, 2H), 3.87 (s, 3H), 2.71 (s, 3H). ^{13}C-NMR (126 MHz, CDCl3) δ: 196.75 159.50, 152.06, 148.57, 141.99, 137.22, 136.91, 133.66, 133.00, 132.24, 129.65, 128.27, 127.05, 125.67, 125.49,114.36, 55.38, 26.93. HRMS (ESI) *m/z* $[M+H]^+$ calcd for $C_{20}H_{18}NO_2$ 304.1332, found 304.1332. 2nd step yield 40**%**
MZ5-3	^{1}H-NMR (500 MHz, CDCl3) δ: 8.45 (d, J = 2.0 Hz, 1H), 8.22 (d, J = 2.0 Hz, 1H), 7.96 (d, J = 8.0 Hz, 1H), 7.87-7.82 (m, 2H), 7.74 (s, 1H), 7.63-7.53 (m, 4H), 6.85 (d, J = 8.5 Hz, 1H), 3.99 (s, 3H), 2.67 (s, 3H). ^{13}C-NMR (126 MHz, CDCl3) δ: 198.01, 163.78, 145.12, 141.47, 141.00, 138.71, 137.70, 137.55, 131.79, 129.82, 129.58, 129.14, 127.46, 126.97, 126.20, 126.15, 125.64, 110.90, 53.59, 26.77. HRMS (ESI) *m/z* $[M+H]^+$ calcd for $C_{20}H_{18}NO_2$ 304.1332, found 304.1333. 2nd step yield 63**%**
MZ5-4	^{1}H-NMR (500 MHz, CDCl3) δ: 9.16 (d, J = 2.0 Hz, 1H), 9.06 d, J = 2.0 Hz, 1H), 8.46 (d, J = 2.0 Hz, 1H), 8.44 (d, J =2.5 Hz, 1H), 7.84 (dd, J = 8.5 Hz, 2.5Hz, 1H), 7.74 (s, 1H), 7.54-7.64 (m, 3H), 6.86 (d, J = 8.5 Hz, 1H), 4.00 (s, 3H), 2.71 (s, 3H). ^{13}C-NMR (126 MHz, CDCl3) δ: 196.65, 163.90, 152.01, 148.73, 145.14, 139.13, 137.54, 137.48, 136.62, 133.65, 132.26, 129.92, 129.44, 126.97, 126.15, 125.60, 111.00, 53.62, 26.92. HRMS (ESI) *m/z* $[M+H]^+$ calcd for $C_{19}H_{17}N_2O_2$ 305.1285, found 305.1282. 2nd step yield 36**%**
MZ5-5	^{1}H-NMR (500 MHz, CDCl3) δ: 8.83 (d, J = 2.0 Hz, 1H), 8.79 (d, J = 2.0 Hz, 1H), 8.23 (d, J = 2.0 Hz, 1H), 8.03 (d, J = 2.5 Hz, 1H), 8.01 d, J = 2.5 Hz, 1H), 7.84 (d, J = 7.5 Hz,1H), 7.57-7.64 (m, 3H), 7.04 (d, J = 9.0 Hz, 2H), 3.88 (s, 3H), 2.68 (s, 3H). ^{13}C-NMR (126 MHz, CDCl3) δ: 197.77, 159.95, 147.19, 146.28,138.47, 137.88, 136.38, 135.63, 132.42, 131.74, 129.83, 129.41, 128.35, 128.07, 126.94, 114.63, 55.39, 26.75. HRMS (ESI) *m/z* $[M+H]^+$ calcd for $C_{20}H_{18}NO_2$ 304.1332, found 304.1327. 2nd step yield 44**%**
MZ5-6	^{1}H-NMR (500 MHz, CDCl3) δ: 9.21 (d, J = 2.0 Hz, 1H), 9.08 (d, J = 2.5 Hz, 1H), 8.89 (d, J = 2.5 Hz, 1H), 8.81 (d, J = 2.0 Hz, 1H), 8.48 (d, J = 2.0 Hz, 1H), 8.04 (d, J = 2.0 Hz, 1H), 7.59 (d, J = 8.5 Hz, 2H), 7.55 (d, J = 8.5 Hz, 2H), 3.89 (s, 3H), 2.73 (s, 3H). ^{13}C-NMR (126 MHz, CDCl3) δ: 196.39, 160.13, 151.86, 149.32, 148.04, 146.08, 136.70, 133.86, 133.77, 132.43, 132.41, 132.37, 129.40, 128.37, 114.72, 55.41, 26.93. HRMS (ESI) *m/z* $[M+H]^+$ calcd for $C_{19}H_{17}N_2O_2$ 305.1285, found 305.1286. 2nd step yield 32**%**
MZ5-7	1H-NMR (500 MHz, CDCl3) δ: 8.85 (d, J = 2.0 Hz, 1H), 8.81 (d, J = 2.0 Hz, 1H), 8.46 (d, J = 2.5 Hz, 1H), 8.21 (d, J = 2.0 Hz, 1H), 8.04-8.00 (m, 2H), 7.88-7.82 (m, 2H), 7.63 (t, J = 8.0 Hz, 1H), 6.90 (d, J = 8.0 Hz, 1H), 4.01 (s, 3H), 2.69 (s, 3H). ^{13}C-NMR (126 MHz, CDCl3) δ: 197.68, 164.30, 147.00, 146.98, 145.29, 138.15, 137.94, 137.41, 135.87, 133.74, 132.43, 131.71, 129.49, 128.25, 126.92, 126.45, 111.35, 53.70, 26.75. HRMS (ESI) *m/z* $[M+H]^+$ calcd for $C_{19}H_{17}N_2O_2$ 305.1285, found 305.1286. 2nd step yield 73**%**
MZ5-8	^{1}H-NMR (500 MHz, CDCl3) δ: 9.22 (d, J = 2.0 Hz, 1H), 9.07 (d, J = 2.5 Hz, 1H), 8.87 (d, J = 2.0 Hz, 1H), 8.85 (d, J = 2.5 Hz, 1H), 8.48 (d, J = 2.5 Hz, 1H), 8.45 (d, J = 3.0 Hz, 1H), 8.02 (d, J = 2.5 Hz, 1H), 7.85 (dd, J = 8.5 Hz, 2.5Hz, 1H), 6.90 (d, J = 8.5 Hz, 1H), 4.01 (s, 3H), 2.73 (s, 3H). ^{13}C-NMR (126 MHz, CDCl3) δ: 196.29, 164.44, 151.83, 149.47, 147.85, 146.81, 145.34, 137.36, 134.08, 133.78, 133.58, 132.68, 132.43, 132.40, 126.07, 111.47, 53.74, 26.93. HRMS (ESI) *m/z* $[M+H]^+$ calcd for $C_{18}H_{16}N_3O_2$ 306.1237, found 306.1233. 2nd step yield 16**%**
