# Supplementary material for: New Series of Zaxinone Mimics (MiZax) for Fundamental and Applied Research
Source: Biomolecules. 2023 Aug 1;13(8):1206. doi: 10.3390/biom13081206 (PMC10452442; doi:10.3390/biom13081206)
Supplement: Supplementary file 1 [file biomolecules-13-01206-s001.zip › Table S2.pdf]

Table S2: Modified Hoagland Nutrient Solution

	Chemicals	mMolar	g/mol	Mass (50xHL) g/L	mL/Litre (+P)	mL/Litre (Low P)
1)	NH_4NO_3	5.6	80.04	22.41	10	10
2)	$K_2HPO_4.3H_2O$	0.4	228.2	5.00	10	0.01
3)	$MgSO_4.7H_2O$	0.8	246.48	9.86	10	10
	K_2SO_4	0.8	174.2	7.00		
4)	$FeSO_4.7H_2O$	0.18	278.0	2.55	10	10
	$Na_2EDTA.2H_2O$		372.2	1.86		
5)	$CaCl_2$	1.6	147.02	11.76	10	10
6)	KNO_3	0.8	101.11	4.04	10	10
7)	**Micronutrients**					
	H_3BO_3	0.023	61.84	0.0711		10
	$MnCl_2.4H_2O$	0.0045	197.91	0.0445		
	$CuSO_4.5H_2O$	0.0003	249.68	0.0037		
	$ZnCl_2$	0.0015	136.32	0.0102		
	$Na_2MoO_4.2H_2O$	0.0001	241.95	0.0012	10	
