# Supplementary material for: New Series of Zaxinone Mimics (MiZax) for Fundamental and Applied Research
Source: Biomolecules. 2023 Aug 1;13(8):1206. doi: 10.3390/biom13081206 (PMC10452442; doi:10.3390/biom13081206)
Supplement: Supplementary file 1 [file biomolecules-13-01206-s001.zip › Table S3.pdf]

Table S3. Primer sequences used in this study

Experiment	Primer name	Sequence (5′–3′)
qRT-PCR	UBQ10-F	TGGTCAGTAATCAGCCAGTTTGG
	UBQ10-R	GCACCACAAATACTTGACGAACAG
	OsD27-RT2-F	CTTCCAAGCTACATCCTCAC
	OsD27-RT2-R	CCCAACCAACCAAGGAAA
	OsCCD7-RT1-F	CAGTCTCCAAGCACAGATG
	OsCCD7-RT1-R	GTTCTTTGGCACCTCTAGTT
	OsCCD8b-RT2-F	TGGCGATATCGATGGTGA
	OsCCD8b-RT2-R	GACCTCCTCGAACGTCTT
	OsMax1-900 qPCR F2	ATTGTCAGCGATCCACTTC
	OsMax1-900 qPCR R2	ATTGTCAGCGATCCACTTC
